# Combining different proteomic approaches to resolve complexity of the milk protein fraction of dromedary, Bactrian camels and hybrids, from different regions of Kazakhstan

**DOI:** 10.1371/journal.pone.0197026

**Published:** 2018-05-10

**Authors:** Alma Ryskaliyeva, Céline Henry, Guy Miranda, Bernard Faye, Gaukhar Konuspayeva, Patrice Martin

**Affiliations:** 1 INRA, UMR GABI, AgroParisTech, Université Paris-Saclay, Jouy-en-Josas, France; 2 INRA, MICALIS Institute, Plateforme d’Analyse Protéomique Paris Sud-Ouest (PAPPSO), Université Paris-Saclay, Jouy-en-Josas, France; 3 CIRAD, UMR SELMET, France; 4 Al-Farabi Kazakh State National University, Biology department, Almaty, Kazakhstan; Centre National de la Recherche Scientifique, FRANCE

## Abstract

Nutritional suitability of milk is not only related to gross composition, but is also strongly affected by the microheterogeniety of the protein fraction. Hence, to go further into the evaluation of the potential suitability of non-bovine milks in human/infant nutrition it is necessary to have a detailed characterization of their protein components. Combining proven proteomic approaches (SDS-PAGE, LC-MS/MS and LC-ESI-MS) and cDNA sequencing, we provide here in depth characterization of the milk protein fraction of dromedary and Bactrian camels, and their hybrids, from different regions of Kazakhstan. A total 391 functional groups of proteins were identified from 8 camel milk samples. A detailed characterization of 50 protein molecules, relating to genetic variants and isoforms arising from post-translational modifications and alternative splicing events, belonging to nine protein families (κ-, α_s1_-, α_s2_-, β-; and γ-CN, WAP, α-LAC, PGRP, CSA/LPO) was achieved by LC-ESI-MS. The presence of two unknown proteins UP1 (22,939 Da) and UP2 (23,046 Da) was also reported as well as the existence of a β-CN short isoform (946 Da lighter than the full-length β-CN), arising very likely in both genetic variants (A and B) from proteolysis by plasmin. In addition, we report, for the first time to our knowledge, the occurrence of a α_s2_-CN phosphorylation isoform with 12P groups within two recognition motifs, suggesting thereby the existence of two kinase systems involved in the phosphorylation of caseins in the mammary gland. Finally, we demonstrate that genetic variants, which hitherto seemed to be species- specific (*e*.*g*. β-CN A for Bactrian and β-CN B for dromedary), are in fact present both in *Camel dromedarius* and *C*. *bactrianus*.

## Introduction

According to the most recent statistics, the world camel population is estimated to be about 29 millions [1]. *Camelus dromedarius* is the most frequent and widespread domestic camel species composing 90% of the total camel population [2]. Camels have been domesticated in a number of arid regions, including Northern and Eastern Africa, the Arabian Peninsula and Central and South West Asia. *Camelus bactrianus* forms numerical inferiority, mostly inhabits in Mongolia, China, and Central Asia. Alternatively, there are also crossed camels (hybrids) which are found mainly in Russia, Iran, Turkmenistan, and in Kazakhstan.

Kazakhstan is a specific region where both domesticated species (*C*. *dromedarius* and *C*. *bactrianus*) along with wild Bactrian camels (*Camelus ferus*) are maintained in mixed herds [3]. There are about 160,000 camel heads reared in this country for milk production [1]. Camel milk is consumed as fresh milk and as a traditional fermented drink called *shubat*, which is very popular in Central Asia countries. Besides nutritional qualities, camel fresh and fermented milk have been reported to display potential health-promoting properties [4–9] which depend very heavily on its unique protein content.

Advanced improvement in proteomic techniques allow nowadays obtaining a precise image of the protein fraction of milk. Recently, proteomic approaches, based on mass spectrometry [10] and isobaric tag for relative and absolute quantification [11], have been used to analyze the proteome of dromedary camel milk and Bactrian camel milk whey, respectively. These techniques were useful to gain knowledge on the detection, quantification and characterization of camel milk proteins. These studies confirm that camel milk is a rich source of biologically active proteins and peptides [12], [13].

Whey proteins which were reported to display a wide range of bioactivities [14], including immuno-modulating [15], anti-carcinogenic [16], antibacterial, and antifungal activities [17], account for 20% of total camel milk proteins. Pattern-recognition proteins, such as the peptidoglycan recognition protein (PGRP), an intracellular component of neutrophils, modulate anti-inflammatory reaction of the immune response [18]. LTF interacts with lipopolysaccharides of Gram-negative bacteria whereas lysozyme C binds and hydrolyzes peptidoglycans, preferably of Gram-positive bacteria, but with a lower affinity than PGRP [19]. Present at a very low level in ruminant milks [20], PGRP has been detected in mammary secretions of porcine and camel [18] and was shown to participate in granule-mediated killing of gram-positive and negative bacteria [21]. Proteose peptone component 3 (PP3 or Lactophorin or GlyCAM1) plays an important immunological role in the lactating camel, to prevent the occurrence of mastitis, or for its newborn by inhibiting pathogen multiplication in the respiratory and gastrointestinal tracts of the suckling young [22]. Likewise, camel milk contains the whey acidic protein (WAP), also found in rodents and lagomorphs [23]. The biological function of this protein is unknown. However, proteins such as elafin and antileukoproteinase 1, containing WAP domains, are known to function as protease inhibitor involved in the immune defence of multiple epithelia and has been identified as candidate molecular markers for several cancers [24].

As in cow milk, *ca*. 80% of the total protein fraction of camel milk are represented by caseins (CN) that are synthesized under multi-hormonal control in the mammary gland of mammals. Associated with amorphous calcium phosphate nanoclusters they form large and stable colloidal aggregates, the so-called CN micelles, which figure as calcium-transport vehicles. These CN micelles provide neonates with calcium at a very high concentration, which is achieved during their packaging in the secretion pathway [25]. Recently it was reported that α_s1_- and α_s2_-CN display molecular chaperone-like activity inhibiting CN aggregation and triggering micelle structure [26].

However, there is no comprehensive investigation on milk protein variations and variability in composition between individual camels. In addition, proteomic studies did not consider the molecular diversity of each type of protein, arising from genetic polymorphisms (mutations), defects in the processing of primary transcripts and post-translational modifications (PTM) such as phosphorylation, factors that significantly have a pronounced impact on protein structure, and finally on milk properties. Milk protein polymorphism is a unique biological paradigm that could help to understand CN intracellular transport, micelle formation and organization, biodiversity and evolution [27], the release of bioactive peptides with implications in human health [28].

Therefore, to gain an insight into the molecular diversity of camel milk proteins, we design a comprehensive strategy combining classical (SDS-PAGE) and advanced proteomic approaches (LC-MS/MS, LC-ESI-MS), as well as cDNA sequencing. Here we report a complete profiling of the milk protein fraction of Bactrian and dromedary camels from Kazakhstan, including a detailed characterization of camel CN and whey proteins including variants related to genetic polymorphisms, splicing defects, phosphorylation levels. In addition, we introduce a reference point for further investigation in camel milk protein polymorphism.

## Materials and methods

### Ethics statements

All animal studies were carried out in compliance with European Community regulations on animal experimentation (European Communities Council Directive 86/609/EEC) and with the authorization of the Kazakh Ministry of Agriculture. Milk sampling was performed in appropriate conditions supervised by a veterinary accredited by the French Ethics National Committee for Experimentation on Living Animals. No endangered or protected animal species were involved in this study. No specific permissions or approvals were required for this study with the exception of the rules of afore-mentioned European Community regulations on animal experimentation, which were strictly followed.

### Milk samples collection and preparation

In total 179 raw milk samples (Table 1) were collected during morning milking on healthy dairy camels belonging to two camel species: *C*. *bactrianus* (n = 72) and *C*. *dromedarius* (n = 65), and their hybrids (n = 42), at different lactation stages, ranging between 30 and 90 days postpartum. Bactrian camels were originating from Kazakh type whereas dromedary camels were from Turkmen Arvana breed. Unfortunately, the information about the nature and the level of hybridization of hybrids was not available. All species are well adapted to the local environment of Kazakhstan.

**Table 1:** Camel milk samples collected (n = 179) in the 3 species of the 4 regions of Kazakhstan.

ID	Region	Coding	Bactrian (B)	Dromedary (D)	Hybrid (H)	Total number of camels for each region
1	Almaty	AL B/D/H	13	20	1	34
2	Shymkent	SH B/D/H	20	21	20	61
3	Kyzylorda	KZ B/D/H	18	16	20	54
4	Atyrau	ZKO B/D/H	21	8	1	30

**Fig 1:**
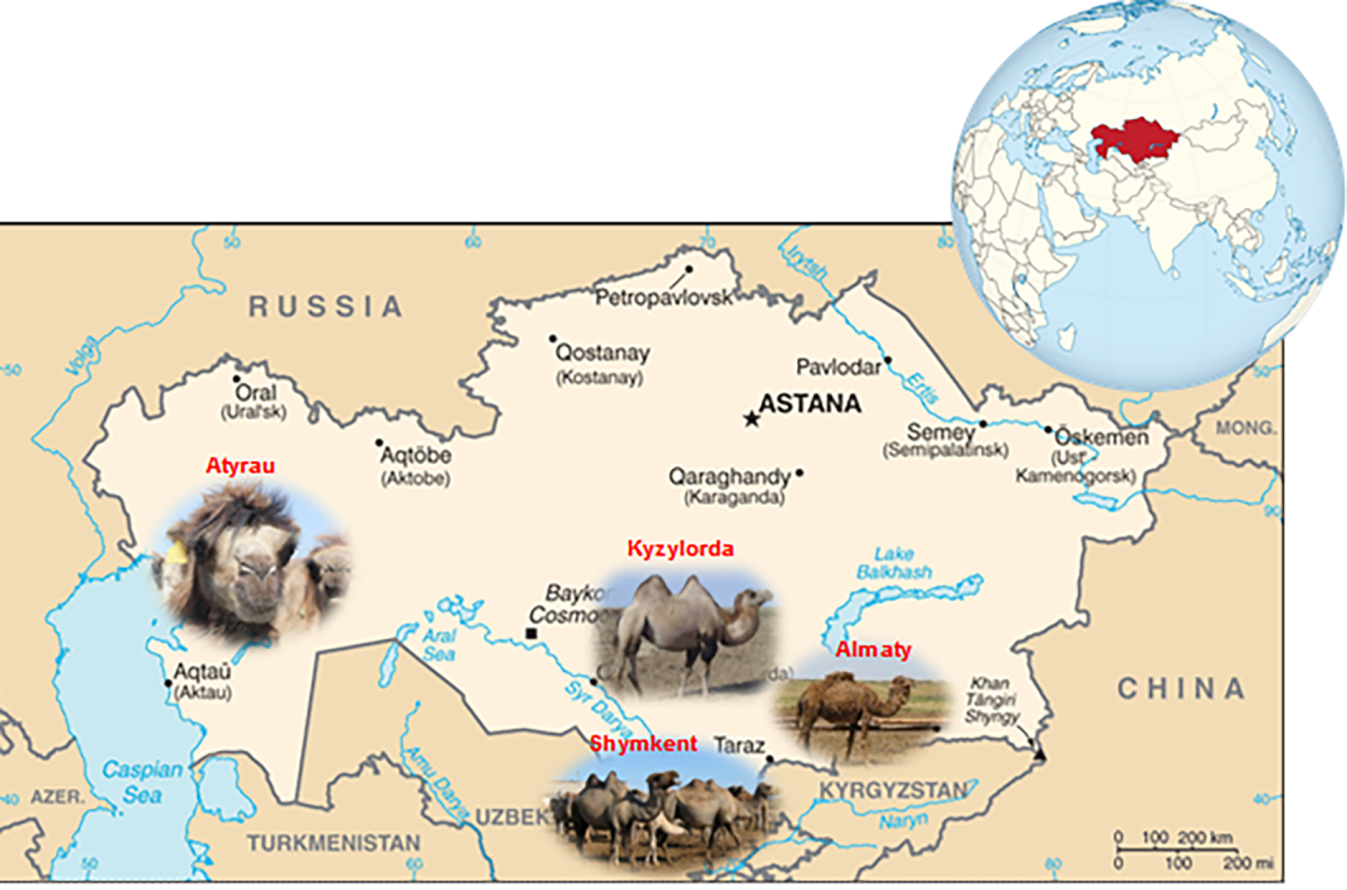
Geographical location of camel milk sampling. Reprinted from http://camelides.cirad.fr/fr/science/pdf/presentation_these_konuspayeva.pdf under a CC BY license, with permission from Konuspayeva Gaukhar, original copyright 2007. https://www.cia.gov/library/publications/the-world-factbook/geos/kz.html https://upload.wikimedia.org/wikipedia/commons/thumb/b/b0/Kazakhstan_on_the_globe_%28Eurasia_centered%29.svg/512px-Kazakhstan_on_the_globe_%28Eurasia_centered%29.svg.png.

Camels grazed on four various natural pastures with the distance more than 3,500 kms between the regions at extreme points of Kazakhstan: Almaty (AL) at the foot of Tien Shan Mountain, Shymkent (SH) along deserts Kyzylkum and Betpak-Dala, Kyzylorda (KZ) on the edge of the steppe, and Atyrau (ZKO) at the mouth of the Caspian Sea (Fig 1). Whole-milk samples were centrifuged at 2,500 *g* for 20 min at 4°C (Allegra X-15R, Beckman Coulter, France) to separating fat from skimmed milk. Samples were quickly frozen and stored at -80°C (fat) and -20°C (skimmed milk) until analysis.

### Selection of milk samples for analysis

**Fig 2:**
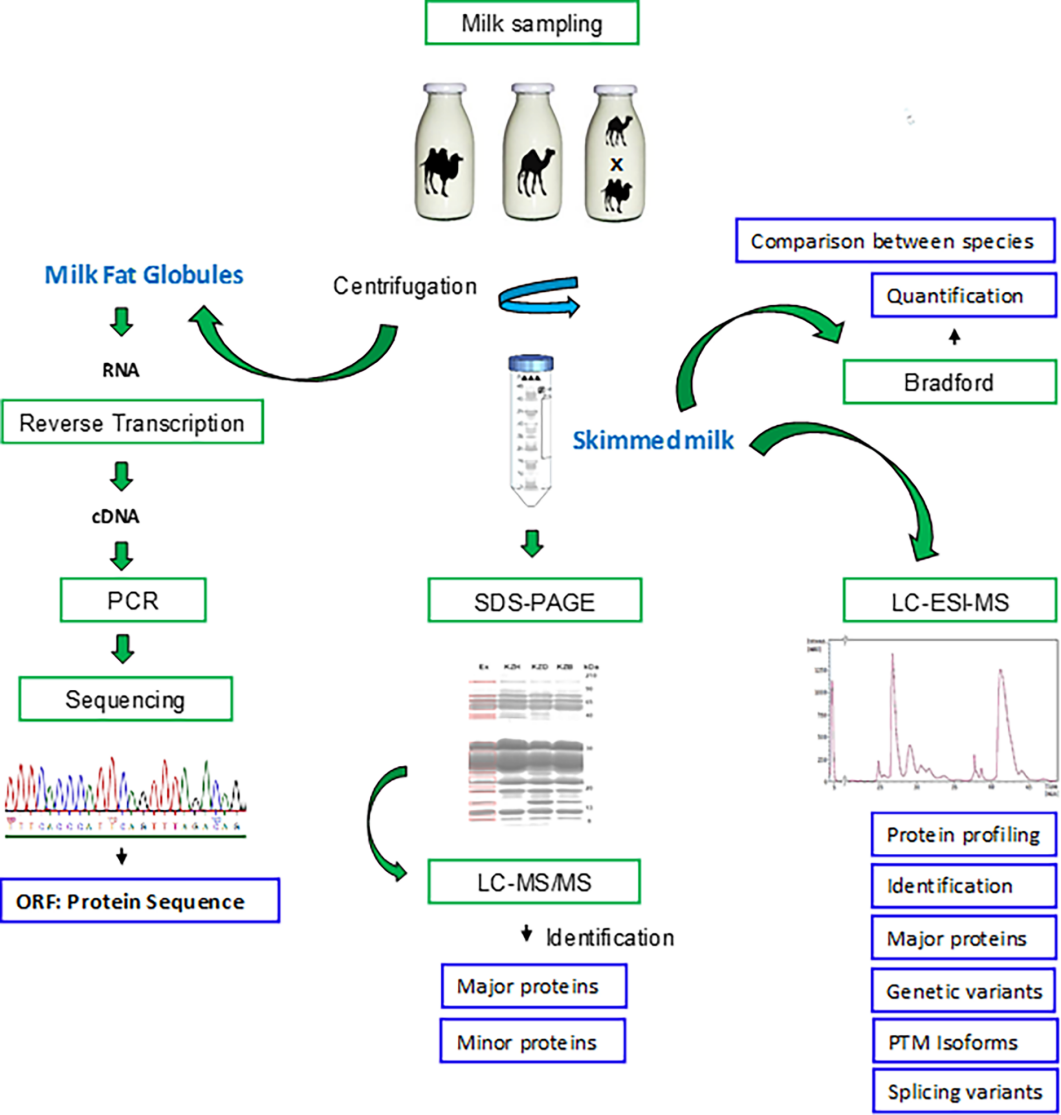
Diagram of the experimental scheme designed for quantification and identification of camel milk proteins.

Of the 179 milk samples collected, 63, including *C*. *bactrianus* (n = 19), *C*. *dromedarius* (n = 20), and hybrids (n = 24) from four different regions of Kazakhstan were selected for SDS-PAGE analysis (Fig 2). Each Bactrian and dromedary camel group formed by 5 animals, except Bactrians of Atyrau regions (n = 4). For hybrids, there were 4 groups comprising 10 animals (Kyzylorda and Shymkent regions), whereas there were only 1 and 3 animals for Almaty and Atyrau regions, respectively. This selection was based on lactation stages and number of parities (from 2 to 14) of each camel group composed by the species and grazing regions. It should be emphasized that data available on animals: breed, age, lactation stage and calving number, were estimated by a local veterinarian, since no registration of camels in farms is maintained. Due to the lack of sufficient information, dromedary milk samples (n = 5) from Almaty region were excluded from subsequent analyses. Then, 8 of the 58 remaining milk samples from three different regions (*C*. *bactrianus*, n = 3, *C*. *dromedarius*, n = 3, and hybrids, n = 2) exhibiting the most representative SDS-PAGE patterns were analyzed by LC-MS/MS after a tryptic digestion of excised gel bands. Additionally, 30 milk samples (*C*. *bactrianus*, n = 10; *C*. *dromedarius*, n = 10; hybrids, n = 10), taken from the 63 milks analyzed by SDS-PAGE, were analyzed by LC-ESI-MS (Bruker Daltonics).

### Coomassie blue (Bradford) protein assay

To estimate the concentration of total protein in a milk sample the Coomassie Blue Protein Assay was used [29]. Absorbance at 590 nm was measured using the UV-Vis spectrophotometer (UVmini-1240, Shimadzu). The reference standard curve was done with commercial bovine serum albumin (BSA) powder dissolved in MilliQ water and diluted to a concentration of 1 mg/mL. Series of dilutions (0.1, 0.2, 0.4, 0.6, and 0.8 μg/μL) were prepared from the stock solution, in duplicate to ensure the protein concentration is within the range of the assay.

### 1D sodium dodecyl sulfate polyacrylamide gel electrophoresis (SDS-PAGE)

Both major and low-abundant proteins resolved by SDS-PAGE were identified after excision by mass analysis of the tryptic hydrolysate. The method used in the study was based on that from Laemmli [30]. Twenty-five micrograms of each individual skimmed milk sample were loaded into 12.5% acrylamide resolving gel and subjected to electrophoresis. Samples were prepared with Laemmli Lysis-Buffer (Sigma-Aldrich). Separations were performed in a vertical electrophoresis apparatus (Bio-Rad, Marnes-la-Coquette, France). After GelCode Blue Safe Protein staining and gel scanning using Image Scanner iii (Epson Expression^TM^ 10,000 XL, Sweden), resolved bands were excised from the gel and submitted to digestion by trypsin. Thereafter, tryptic peptides were analyzed by LC-MS/MS.

### Identification of proteins by LC-MS/MS analysis

In order to identify the main protein contained in each electrophoretic band, mono dimensional electrophoresis (1D SDS-PAGE) followed by trypsin digestion and by LC-MS/MS analysis, was used essentially as described [31]. Briefly, after a 10 cm migration of samples in such an 1D SDS-PAGE, the 16 main electrophoretic bands (1.5 mm^3^) were cut on each gel lane, transferred into 96-well microtiter plates (FrameStar, 4titude, 0750/Las). Reduction of disulfide bridges of proteins was carried out by incubating at 37°C for one hour with dithiothreitol (DTT, 10 mM, Sigma), meanwhile the alkylation of free cysteinyl residues with iodoacetamide (IAM, 50 mM, Sigma) at room temperature for 45 min in total obscurity. After gel pieces were washed twice, first, with 100 μL 50% ACN/50 mM NH_4_HCO_3_ and then with 50 μL ACN, they were finally dried. The hydration was performed at 37°C overnight using digestion buffer 400 ng lys-C protease + trypsin. Hereby, peptides were extracted with 50% ACN/0.5% TFA and then with 100% ACN. Peptide solutions were dried in a concentrator and finally dissolved into 70 μL 2% ACN in 0.08% TFA.

The identification of peptides was obtained using UltiMate™ 3000 RSLCnano System (Thermo Fisher Scientific) coupled either to LTQ Orbitrap XL™ Discovery mass spectrometer or QExactive (Thermo Fischer Scientific).

Four μL of each sample was injected with flow of 20 μL/min on a precolumn cartridge (stationary phase: C18 PepMap 100, 5 μm; column: 300 μm x 5 mm) and desalted with a loading buffer 2% ACN and 0.08% TFA. After 4 min, the precolumn cartridge was connected to the separating RSLC PepMap C18 column (stationary phase: RSLC PepMap 100, 2 μm; column: 75 μm x 150 mm). Elution buffers were A: 2% ACN in 0.1% formic acid (HCOOH) and B: 80% ACN in 0.1% HCOOH. The peptide separation was achieved with a linear gradient from 0 to 35% B for 34 min at 300 nL/min. One run took 42 min, including the regeneration and the equilibration steps at 98% B.

Peptide ions were analyzed using Xcalibur 2.1 with the following machine set up in CID mode: 1) full MS scan in Orbitrap with a resolution of 15 000 (scan range [m/z] = 300–1600) and 2) top 8 in MS/MS using CID (35% collision energy) in Ion Trap. Analyzed charge states were set to 2–3, the dynamic exclusion to 30 s and the intensity threshold was fixed at 5.0 x 10^2^.

Raw data were converted to m*z*XML by MS convert (ProteoWizard version 3.0.4601). UniProtKB Cetartiodactyla database was used (157,113 protein entries, version 2015), in conjunction with contaminant databases were searched by algorithm X!TandemPiledriver (version 2015.04.01.1) with the software X!TandemPipeline (version 3.4) developed by the PAPPSO platform (http://pappso.inra.fr/bioinfo/). The protein identification was run with a precursor mass tolerance of 10 ppm and a fragment mass tolerance of 0.5 Da. Enzymatic cleavage rules were set to trypsin digestion (“after R and K, unless P follows directly after”) and no semi-enzymatic cleavage rules were allowed. The fix modification was set to cysteine carbamido methylation and methionine oxidation was considered as a potential modification. Results were filtered using inbuilt X!TandemParser with peptide *E*-value of 0.05, a protein *E*-value of -2.6, and a minimum of two peptides.

### LC-ESI-MS

Fractionation of camel milk proteins and determination of their molecular masses, performed by coupling RP-HPLC to ESI-MS (micrOTOF^TM^ II focus ESI-TOF mass spectrometer; Bruker Daltonics), were essentially as described [31]. In total 20 μL of skimmed milk samples were first clarified by the addition of 230 μL of clarification solution 0.1 M bis-Tris buffer pH 8.0, containing 8 M urea, 1.3% trisodium citrate, and 0.3% DTT. Clarified milk samples (25 μL) were directly injected onto a Biodiscovery C5 reverse phase column (300 Å pore size, 3 μm, 150 x 2.1 mm; Supelco, France). The mobile phase of the column corresponded to a gradient mixture of Solvent A (H_2_O/TFA 100:0.25, v/v) and Solvent B (ACN/TFA 100:0.20, v/v). Elution was achieved using a linear gradient from 5% to 27% B in 20 min, from 27% to 33% B in 0.1 min, from 33% to 34% B in 11.1 min, from 34% to 40% B in 0.1 min, from 40% to 41% B in 14.9 min, and from 41% to 90% B in 0.1 min. This gradient elution was followed by an isocratic elution at 90% B for 4.9 min, and a linear return to 5% B in 0.1 min. The temperature of the column was adjusted to 52°C and the flow rate to 0.2 mL/min. Eluted peaks were detected by UV-absorbance at 214 nm. The liquid effluent was introduced to the mass spectrometer. Positive ion mode was used, and mass scans were acquired over a mass-to-charge ratio (m/z) ranging between 600 and 3000 Da.

The LC/MS system was controlled by the HyStar software (Bruker Daltonics). Peak profiles from UV 214 nm and Extracted Ion Chromatograms (EIC), multicharged ion spectra, deconvoluted spectra and determination of masses were obtained with DataAnalysis Version 4.0 SP1 software (Bruker Daltonics).

### Milk fat globule collection and RNA extraction

Milk was centrifuged at 2,500 *g* for 20 min to pellet somatic cells (SC) and to separate the upper milk fat globule (MFG) fraction. The MFG fraction was mixed with Trizol LS and heated briefly at 30°C while shaking, to emulsify fat. Total RNA was extracted from milk fat using Trizol (Invitrogen) following the protocol from the manufacturer, as described in Brenaut *et al*. [32].

### First-strand cDNA synthesis and PCR amplification

First-strand cDNA was synthesized from 5 to 10 ng of total RNA primed with oligo(dT)20 and random primers (3:1, vol/vol) using Superscript III reverse transcriptase (Invitrogen Life Technologies Inc., Carlsbad, CA) according to the manufacturer’s instructions. One microliter of 2 U/μL RNase H (Invitrogen Life Technologies) was then added and the reaction mix was incubated for 20 min at 37°C to remove RNA from heteroduplexes. Single-strand cDNA thus obtained was stored at -20°C. cDNA samples covering the entire coding regions of caseins were amplified. PCR was performed in an automated thermocycler GeneAmp® PCR System 2,400 (Perkin-Elmer, Norwalk, USA) with GoTaq® G2 Flexi DNA Polymerase Kit (Promega Corporation, USA). Reactions were carried out with 0.2 mL thin-walled PCR tubes with flat cap strips (Thermo Scientific, UK), in 50 μL volumes containing 5X Green or Colorless GoTag® Flexi Buffer, MgCl_2_ Solution 25 mM, PCR Nucleotide Mix 10 mM each, GoTag® G2 Flexi DNA Polymerase (5 U/μL), 10 mM each oligonucleotide primer, template DNA and nuclease-free water, up to the final volume. Primer pairs, purchased from Eurofins (Eurofins genomics, Germany), were designed using published *Camelus* nucleic acid sequence. Sequencing of PCR fragments was performed with primer pairs used for PCR and sequenced from both strands, according to the Sanger method by Eurofins.

## Results

### Total protein content

Using the Bradford assay for estimating the protein concentration in milk samples, we observed that the highest protein concentration occurred with Bactrian camel milk samples, but the difference was slight comparing with crossed camel species. The total protein value in raw camel milk from Shymkent region was estimated to be *ca*. 33 g/L (33.15 ± 6.64 g/L) for *C*. *bactrianus* (n = 5), and 31 g/L (30.83 ± 5.82 g/L) for *C*. *dromedarius* (n = 7), whereas hybrids (n = 9) displayed an intermediate value 31.5 g/L (31.43 ± 4.56 g/L). On average, Bactrian milk was considered to have a higher total protein content than that of Dromedary [33] and hybrid milks. Our results are in agreement with data reported previously by Konuspayeva *et al*. [34]. No significant differences were found across species from different geographical locations.

### Identification of main milk proteins from 1D SDS-PAGE by LC-MS/MS

**Fig 3:**
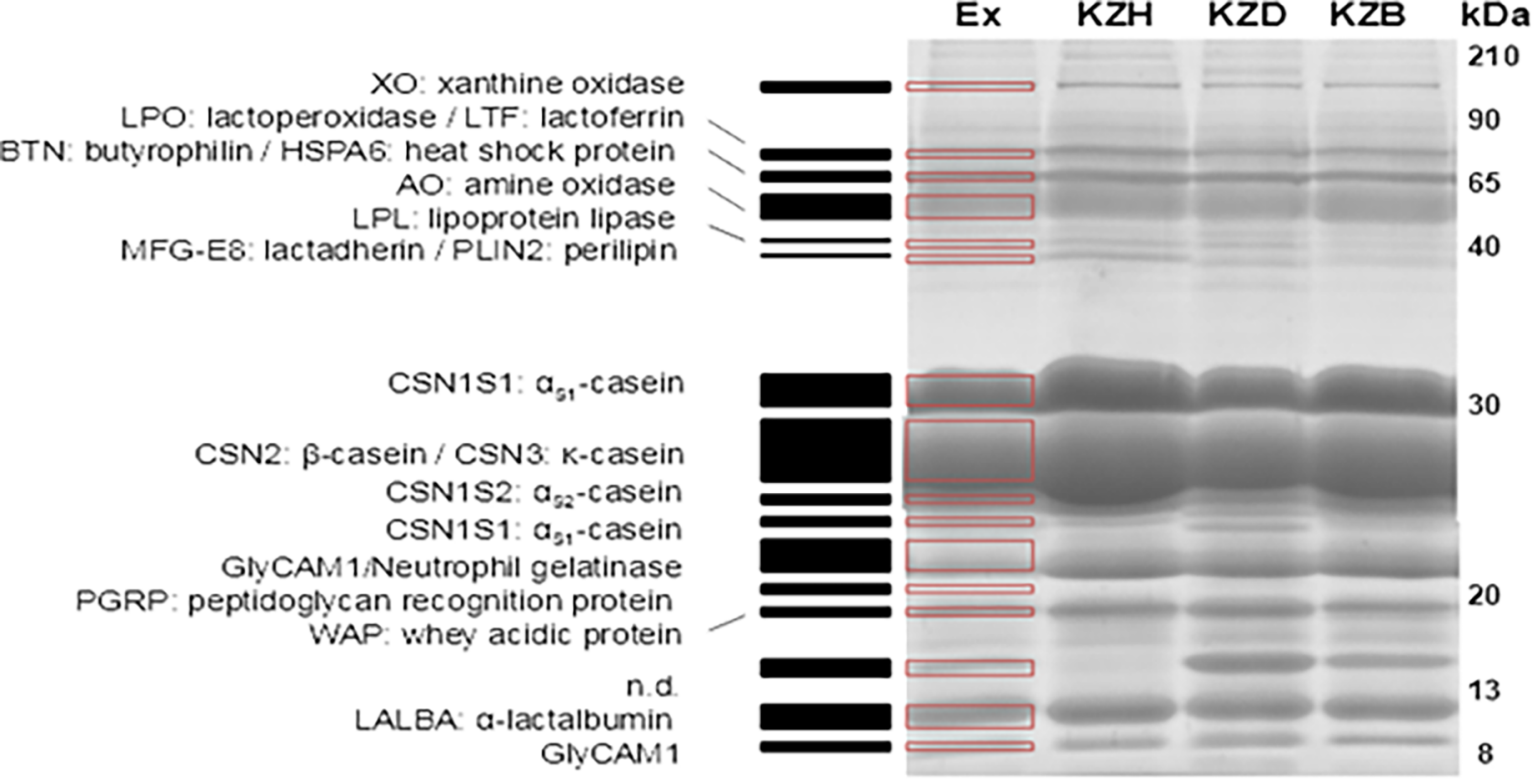
1D SDS-PAGE pattern of *C*. *bactrianus* (KZB), *C*. *dromedarius* (KZD) and hybrid (KZH) skimmed milk samples of Kyzylorda (KZ) region. Red frames and black boxes aligned correspond to electrophoretic bands that were excised from the gel and subsequently analyzed for protein identification, after tryptic digestion, by LC-MS/MS. Molecular weight markers from 210 to 8 kDa are indicated at the right of the gel.

After first adjusting protein concentrations at the same value, 63 individual camel milk samples were separated onto SDS-PAGE. The comparative analysis of whole milk samples by SDS-PAGE displayed rather similar electrophoretic profiles with related migration characteristics and the same apparent molecular weights between individual milk samples of different species and regions. A typical gel pattern from which proteins were identified in individual *C*. *bactrianus*, *C*. *dromedarius* and hybrid milk samples of Kyzylorda region is shown in Fig 3.

Sixteen main bands relatively well-resolved were excised from the electrophoretic pattern. The most intense band observed around 26 kDa was identified as β-CN. Quantitative analyses on camel milk proteins carried out before have demonstrated significantly higher amounts of β-CN compared to the homologous bovine CN [35]. The most representative other bands were characterized as being: WAP (12.5 kDa), α-LAC (14.3 kDa), GlyCAM 1 (15.4 kDa and 17.2 kDa), κ-CN (20.3 kDa), PGRP (21.3 kDa), α_s2_-CN (22.9 kDa), α_s1_-CN (25.7 kDa), neutrophil gelatinase (28.3 kDa), lipoprotein lipase (46.5 kDa), perilipin-2 (47.2 kDa), butyrophilin (51.0 kDa), amine oxidase (55.3 kDa), lactadherin (56.2 kDa), heat shock protein (70.0 kDa), LTF (77.1 kDa), lactoperoxidase (87.7 kDa), and xanthine oxidase (150 kDa). Masses mentioned above correspond to theoretical masses of proteins identified on the basis of tryptic profiles after LC-MS/MS analysis. Globally, the electrophoretic patterns of Kazakh camel milk samples agree with those reported recently for Israelian and Tunisian camel milk samples [36], [37]. However, surprisingly the prominent fact was the apparent absence in Kazakh milk samples of camel serum albumin (CSA), the major whey protein with a molecular mass equal to 66.0 kDa in camel colostrum [36]. By contrast, this protein has been successfully identified, with the best *E*-value, in Tunisian fresh milk samples [37].

### Qualitative proteome of camel skimmed milk by LC-MS/MS

We took advantage of LC-MS/MS analysis to identify proteins in electrophoretic bands to go further into the description of the protein fraction of camel milk. Indeed, for each band analyzed by LC-MS/MS, between 10 and 70 different proteins were identified. In such a way, using UniprotKB taxonomy cetartiodactyla (SwissProt + Trembl) database, a total of 391 functional groups of proteins (proteins belonging to a same group share common peptides) were identified after LC-MS/MS analysis of 8 camel milk samples (S1 Table). A set of 235 proteins was observed as common to the 8 milk samples. As example, a list of the first 70 common proteins found in milk samples of the three species from Shymkent region is given in Table 2.

**Table 2:** Top 70 proteins identified by LC-MS/MS from individual *C*. *bactrianus* (B), *C*. *dromedarius* (D) and hybrid (H) milk samples of Shymkent region.

ID	Accession	Description	Mr	(-) log *E*-value			Coverage, %			Number of Spectra*		
			kDa	B	D	H	B	D	H	B	D	H
1	O97943-2	Short isoform of Alpha-S1-casein *(C*. *dromedarius)*	25,7	453,63	535,3	585,79	88	92	92	574	679	842
2	A0A077SL35	Beta-casein *(C*. *bactrianus)*	26,1	299,05	285,89	395,72	77	79	79	542	507	745
3	O97944	Alpha-S2-casein *(C*. *dromedarius)*	22,9	285,16	320,65	276,39	70	70	66	357	451	376
4	W6GH05	Lactoferrin *(C*. *dromedarius)*	77,1	723,25	557,5	1174,16	85	80	88	253	157	829
5	P15522-2	Isoform B of Glycosylation-dependent cell adhesion molecule 1 *(C*. *dromedarius)*	15,4	112,39	189,45	192,31	64	70	69	220	359	358
6	L0P304	Kappa-casein *(C*. *bactrianus)*	20,3	127,64	149,66	194,03	51	53	51	217	222	329
7	S9WF76	Lactadherin-like protein *(C*. *ferus)*	45,6	263,03	350,07	501,73	51	53	57	162	236	436
8	P00710	Alpha-lactalbumin *(C*. *dromedarius)*	14,3	288,2	277,9	329,27	83	80	83	161	159	175
9	Q9GK12	Peptidoglycan recognition protein 1 *(C*. *dromedarius)*	21,3	308,94	359,49	336,8	73	73	79	112	156	140
10	S9Z0L8	Amine oxidase [flavin-containing] *(C*. *ferus)*	55,3	233,97	231,94	277,32	74	70	75	110	138	190
11	S9Y4T1	Xanthine dehydrogenase/oxidase *(C*. *ferus)*	150	270,07	435,86	330,26	40	45	41	79	138	121
12	S9X3X3	Butyrophilin subfamily 1 member A1 *(C*. *ferus)*	51,09	95,37	140,19	162,3	47	50	51	61	107	111
13	S9X4G0	Neutrophil gelatinase-associated lipocalin-like protein *(C*. *ferus)*	28,3	104,88	98,15	165,14	48	45	62	44	33	82
14	S9X1L5	Lipoprotein lipase isoform 3 (Fragment) *(C*. *ferus)*	46,5	107,66	133,72	54,73	43	55	27	43	56	29
15	S9YK74	Perilipin *(C*. *ferus)*	47,2	116,47	169,78	167,59	56	60	52	39	67	46
16	P09837	Whey acidic protein *(C*. *dromedarius)*	12,5	42,97	59,92	82,11	55	75	84	36	43	73
17	S9X4X6	Uncharacterized protein *(C*. *ferus)*	43,1	47,87	14	17,5	30	13	16	36	9	16
18	S9X7Q1	Lactoperoxidase isoform 1 preproprotein *(C*. *ferus)*	87,7	83,1	62,59	65,16	26	22	20	25	19	17
19	S9XDK9	Complement C3-like protein *(C*. *ferus)*	262,7	87,48	44,5	292,06	12	4	24	25	8	69
20	S9XR87	Beta-2-microglobulin *(C*. *ferus)*	14,8	36,87	36,15	40,76	43	43	43	23	28	44
21	O18831	Growth/differentiation factor 8 *(S*. *scrofa)*	42,7	44,62	49,29	67,44	28	24	32	23	22	17
22	P68103	Elongation factor 1-alpha 1 *(B*. *taurus)*	50	58,23	42,59	78,3	31	27	31	20	12	24
23	S9YCI6	Peptidyl-prolyl cis-trans isomerase *(C*. *ferus)*	23,8	44,54	43,1	71,24	54	54	57	20	21	22
24	S9XP75	Monocyte differentiation antigen CD14 *(C*. *ferus)*	29,7	49,75	44,58	73,91	27	27	27	17	16	22
25	S9WCV2	Sulfhydryl oxidase *(C*. *ferus)*	72,2	31,64	76,49	14,43	18	27	8	16	26	6
26	S9YC53	Alpha-1-antitrypsin-like protein *(C*. *ferus)*	51,9	64,76	64,84	51,84	28	31	32	14	21	15
27	S9YS49	Putative E3 ubiquitin-protein ligase Roquin *(C*. *ferus)*	158,5	56,65	90,28	8,84	10	14	2	14	29	3
28	T0NN97	Uncharacterized protein *(C*. *ferus)*	151,4	38,86	53,95	36,72	12	13	11	13	16	15
29	A0A0F6YEF6	Anti-HCV NS3/4A serine protease immoglobulin heavy chain (Fragment) *(C*. *dromedarius)*	13,4	38,24	35	52,95	24	24	24	12	10	26
30	S9X9X0	Vitelline membrane outer layer protein 1-like protein *(C*. *ferus)*	24,2	44,94	52,11	75,95	51	50	53	12	14	31
31	S9X358	Tissue alpha-L-fucosidase *(C*. *ferus)*	42,7	25,83	23,27	24,46	23	18	18	12	12	7
32	S9WS72	Sodium-dependent phosphate transport protein 2B-like protein *(C*. *ferus)*	75,4	24,99	15,41	24,7	18	7	9	11	8	8
33	S9X2V0	UDP-Gal:betaGlcNAc beta 1,4-galactosyltransferase 1, membrane-bound form-like protein *(C*. *ferus)*	37,3	30,18	13,09	38,99	23	11	33	11	7	15
34	S9XSQ6	Vitamin D-binding protein-like protein *(C*. *ferus)*	49,3	41,66	109,25	18,01	24	45	8	10	30	3
35	S9WGZ9	Rab GDP dissociation inhibitor beta isoform 1 *(C*. *ferus)*	35,7	27,75	22,32	55,78	32	25	43	10	8	15
36	P19120	Heat shock cognate 71 kDa protein *(B*. *taurus)*	71,1	37,14	60,92	111,21	17	24	35	9	16	30
37	S9XA25	Ezrin isoform 5-like protein (Fragment) *(C*. *ferus)*	71	24,32	28,23	47,46	13	10	20	9	7	13
38	S9XI30	Uncharacterized protein *(C*. *ferus)*	22	25,16	66,23	20,66	38	54	39	9	31	8
39	S9Y2X0	Platelet glycoprotein 4 *(C*. *ferus)*	38,3	28,14	40,27	55,15	15	17	29	9	8	17
40	G9F6X8	Protein disulfide-isomerase *(S*. *scrofa)*	56,3	20,09	20,62	21,76	17	15	9	9	10	3
41	S9WZP7	Serpin A3-8 *(C*. *ferus)*	74,9	15,26	28,06	72,32	11	12	20	8	12	28
42	A7MBJ4	Receptor-type tyrosine-protein phosphatase F *(B*. *taurus)*	211,1	23,69	21,19	20,54	6	6	4	8	8	8
43	S9YFG2	Complement factor D *(C*. *ferus)*	38,1	37,99	33,7	44,44	14	14	14	8	9	13
44	S9WPL9	Uncharacterized protein *(C*. *ferus)*	84,6	31,09	39,23	57,64	12	10	17	7	6	18
45	S9XE02	Beta-2-glycoprotein 1-like protein *(C*. *ferus)*	30,9	46,22	64,63	48,09	42	43	27	7	12	5
46	B8XH67	Na(+)/H(+) exchange regulatory cofactor NHE-RF *(S*. *scrofa)*	39,2	20,94	37,33	32,94	22	22	30	7	9	11
47	Q28452	Quinone oxidoreductase *(L*. *guanicoe)*	35,1	23,01	24,97	51,04	28	25	43	7	5	11
48	S9YU13	Glutathione S-transferase-like protein *(C*. *ferus)*	27,7	34,68	8,59	22	21	15	19	7	3	4
49	P00727-2	Isoform 2 of Cytosol aminopeptidase *(B*. *taurus)*	53,9	14,58	13,07	35,48	15	9	18	6	4	7
50	S9WUC8	Ig kappa chain V-II region RPMI 6410-like protein *(C*. *ferus)*	26,7	22,72	12,39	32,25	19	10	27	6	4	15
51	S9WRY0	L-lactate dehydrogenase B chain isoform 1-like protein *(C*. *ferus)*	30,2	25,51	28,95	37,66	22	22	22	6	7	6
52	S9X5V9	Fc of IgG binding protein (Fragment) *(C*. *ferus)*	254,9	20,45	65,48	10,4	3	9	2	6	16	4
53	B5B0D4	Major allergen beta-lactoglobulin *(B*. *taurus)*	19,8	9,94	54,67	12,3	21	59	25	6	27	7
54	S9XE13	Uncharacterized protein *(C*. *ferus)*	82,6	12,16	32,28	71,26	5	10	15	5	17	22
55	S9Y8C6	Phosphoglucomutase 1 isoform 3-like protein *(C*. *ferus)*	68,3	11,37	18,27	42,46	9	11	22	5	6	11
56	S9Y3S5	Olfactory receptor *(C*. *ferus)*	108,3	19,83	37,9	43,59	4	6	7	5	9	11
57	S9XT33	Lipopolysaccharide-binding protein *(C*. *ferus)*	47,5	11,05	9,43	16,89	12	10	17	5	4	7
58	S9YL21	Apolipoprotein A-I *(C*. *ferus)*	22,5	19,05	68,11	82,98	27	49	57	5	12	14
59	S9Y5X2	Cell death activator CIDE-A-like protein *(C*. *ferus)*	41,2	16,09	22,41	15,11	11	14	10	4	11	4
60	S9XC74	Osteopontin isoform OPN-c *(C*. *ferus)*	34,6	8,45	12,77	34,67	8	11	23	4	10	24
61	T0NLV9	Epoxide hydrolase 1 *(C*. *ferus)*	54,3	7,1	11,53	12,54	9	14	10	4	6	4
62	S9WKD1	Ribonuclease 4 *(C*. *ferus)*	26,8	11,56	25,5	29,63	12	22	23	3	7	9
63	S9WDV3	Fibrinogen gamma chain isoform gamma-B *(C*. *ferus)*	50,5	7,22	33,1	76,88	6	49	37	3	12	33
64	S9XLJ3	Brain-specific serine protease 4-like protein *(C*. *ferus)*	44,6	7,52	8,26	9,3	10	10	12	3	3	4
65	S9WY98	Sodium/glucose cotransporter 1 *(C*. *ferus)*	78,3	9,01	5,72	14,27	4	3	5	3	2	5
66	S9WX48	Alpha-1-acid glycoprotein *(C*. *ferus)*	22,9	6,65	7,25	29,92	14	8	44	3	2	17
67	W5P9V5	Uncharacterized protein *(O*. *aries)*	85,3	7,14	7,74	8,54	3	3	3	3	3	3
68	T0NLF0	Vitronectin *(C*. *ferus)*	56,2	11,18	7,8	25,19	7	4	10	3	2	4
69	Q0IIG8	Ras-related protein Rab-18 *(B*. *taurus)*	22,9	3,49	15,33	21,19	10	28	24	2	5	4
70	S9YNY9	Nucleobindin-1 *(C*. *ferus)*	53	4,54	48,27	20,28	5	41	21	2	16	9

Molecular masses (M_r_) of proteins are expressed in kDa, E-value in log, coverage in %. Spectra indicates the number of spectra permitting the identification of proteins. Major proteins identified in excised gel bands after SDS-PAGE are given in bold type.

*abundance of each protein was estimated from spectral count. The number of spectra of C. bactrianus (B) classified the table.

Eight proteins were identified as authentically matching with proteins in *C*. *dromedarius* protein database, two with *C*. *bactrianus* protein database, 46 with *C*. *ferus* protein database, and the remaining (n = 14) with the other mammalian species such as, *Lama guanicoe*, *Bos taurus*, *Sus scrofa* and *Ovis aries* protein databases. Immune-related proteins such as GlyCAM1, lactadherin (MFG-E8), and LTF, as well as milk fat globule membrane (MFGM)-enriched proteins such as xanthine oxidase (XO), butyrophilin (BTN), actin, ras-related protein Rab-18, ADP-ribosylation factor 1, tyrosine-protein kinase and GTP-binding protein SAR1b, were detected. Likewise, proteins originating from blood such as serpin A3-1, apolipoprotein A-1, α-1-antitrypsin like protein, α-1-acid glycoprotein, β-2-microglobulin, complement C3-like protein were found in all milk samples analyzed.

### Camel milk protein profiling by LC-ESI-MS

Thirty individual milk samples, including *C*. *bactrianus* (n = 10), *C*. *dromedarius* (n = 10), and hybrids (n = 10) taken from the 58 milk samples analyzed in SDS-PAGE were submitted to LC-ESI-MS analysis. Milk proteins separated by RP-HPLC were identified based on their molecular mass, arising from ESI-MS. Putative genetic variants and post-translational (glycosylation and phosphorylation) isoforms were determined by deconvoluting multiple charged ion spectra in a real mass scale. Knowing their primary structures, it is possible to determine molecular masses of non post-translationally modified proteins, and then we can precisely know the mass of phosphorylation isoforms resulting from the addition of phosphate groups (±79.98 Da). Likewise, masses of isoforms arising from cryptic splice site usage, usually leading to the loss of the first codon (CAG) of an exon specifying a glutaminyl residue (-128 Da), are easily deduced. A camel mass reference database was thus created for the main milk proteins by combining the data available from *C*. *dromedarius*, *C*. *bactrianus*, *C*. *ferus*, *and Lama glama* milk protein sequences published in UniProtKB (ExPASy SIB Bioinformatics Resource Portal) and the National Centre for Biotechnology Information (NCBI).

**Fig 4:**
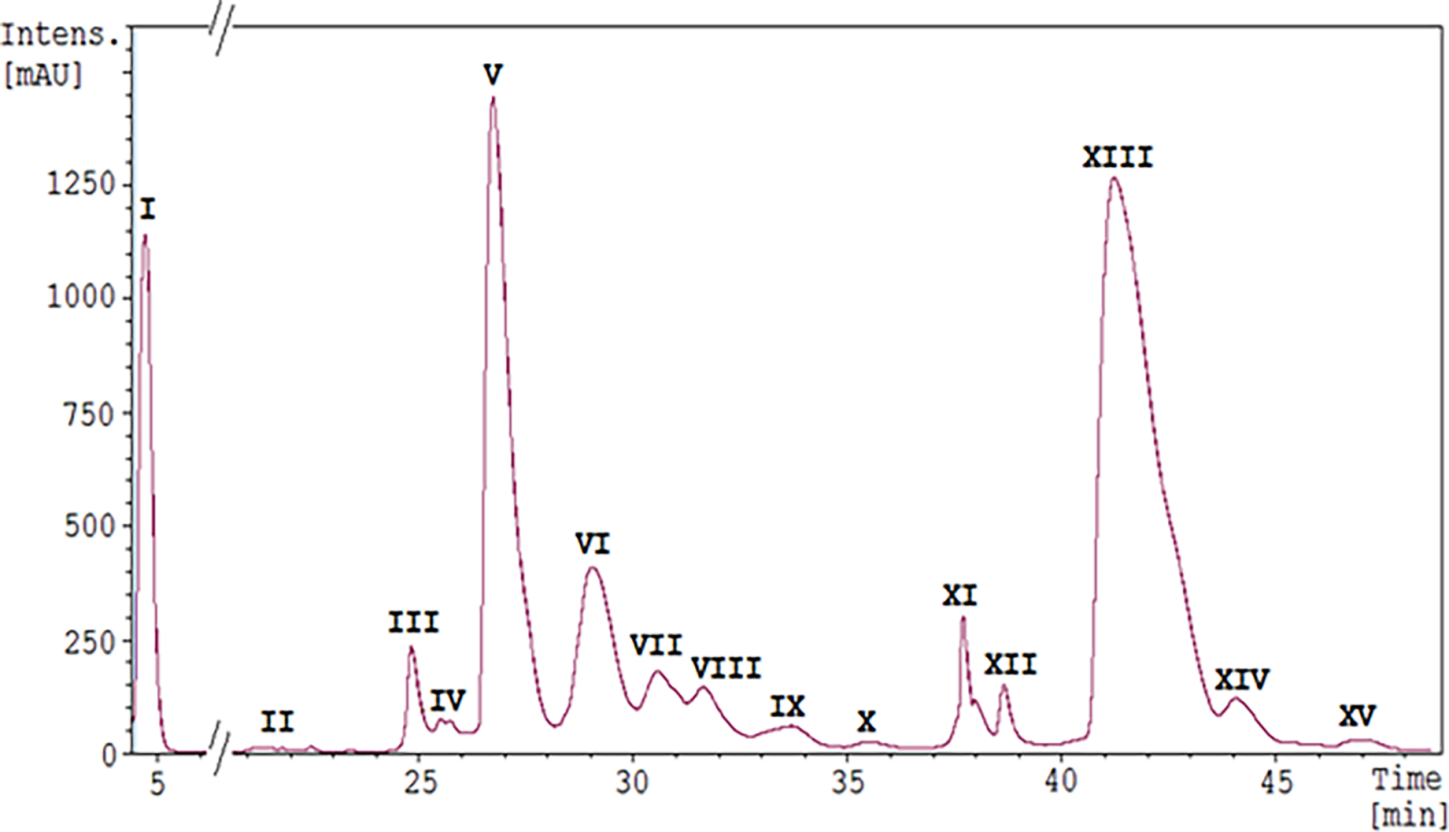
LC-ESI-MS profile of clarified crossed camel milk of Kyzylorda region. Nine major milk protein fractions were identified in the following order: peak I and II contained glycosylated ant natural isoforms of κ-CN; peak III: WAP; peaks IV, V: α_s1_-CN; peak VI: α-LAC, α_s1_-CN and UP1; peak VII: α_s2_-CN and UP1; peaks VIII, IX, and X α_s2_-CN along with UP2 in peak X; peak XI: PGRP and UP2; peak XII: CSA/LPO; peaks XIII and XIV: β-CN, and peak XV: γ_2_-CN.

To illustrate the efficiency of such an approach, a typical protein profile obtained with a milk from a hybrid camel sampled in Kyzylorda region is given in Fig 4. The analysis of molecular isoforms, identified from mass data, are reported in Table 3, in which experimental and theoretical molecular masses of camel milk proteins are given and confronted. The mass accuracy has allowed distinguishing about 50 protein molecules corresponding to isoforms belonging to nine protein families, eluted from the reverse-phase column as 15 peaks.

In peak I, the two molecular masses (21,157 Da and 21,184 Da) found were associated with glycoforms of κ-CN. The molecular mass of 21,157 Da corresponds to mono-phosphorylated variant A of κ-CN with tri-saccharides ((GaN-Ga-SA2) x 3 or (GaN-Ga) + (GaN-Ga-SA3) x 2, or (GaN-Ga-SA) + (GaN-Ga-SA2) + (GaN-Ga-SA3)). The molecular mass of 21,184 Da was expected to be non-phosphorylated variant B of κ-CN with penta-saccharides ((GaN-Ga) x 3 + (GaN-Ga-SA2) x 2, or (GaN-Ga) + (GaN-Ga-SA) x 4, or (GaN-Ga) x 2 + (GaN-Ga-SA) x 2 + (GaN-Ga-SA2), or (GaN-Ga) x 3 + (GaN-Ga-SA) + (GaN-Ga-SA3)). Peak II contained molecules of which the molecular masses (18,210 Da and 18,236 Da) were identified as non-phosphorylated variant B of κ-CN along with the A variant modified at its N-terminal residue to form a pyro-glutamic acid (pyro-E), which is formed spontaneously by cyclization of the N-terminal E residue. The two molecular masses: 12,564 Da and 12,644 Da, detected in peak III, were assigned to the WAP peptide chain without or with one P group, respectively. Peaks IV, V, and VI were shown to contain α_s1_-CN. The molecular mass of 23,878 Da observed in peak IV was interpreted as being a short isoform (201-residues) of α_s1_-CN variant A with 4P groups, arising from exons 13’ and 16 skipping events in the mature mRNA during the course of primary transcripts splicing, resulting in deleted sequences (residues E112-Q117 and E155-E162). Despite identification of only one splicing isoform with 4P groups (23,878 Da) in this milk sample, isoforms with 3P and 5P, along with cryptic splice site usage were identified in several other milk samples. Peak V consisted of three relative groups of three masses with sequential increments (s.i.) of 80 Da: 24,547 Da—24,707 Da, 24,675 Da—24,835 Da, and 24,689 Da—24,849 Da. The mass difference (128 Da) between the first and the second group (Table 3) corresponds to the loss of glutaminyl residue 83 (ΔQ83), encoded by the first codon (CAG) of exon 11. As reported previously [38], 24,755 Da was identified as the short isoform (207-residues) of the α_s1_-CN variant A originating from exon 16 skipping during the course of the primary transcript processing. The mass difference (14 Da) between the second (24,675 Da) and the third (24,689 Da) group is due to the aa substitution E30D reported by Shuiep *et al*. [39] characterizing the C variant. Thus, it is concluded that the third mass group gathers α_s1_-CN short isoforms (207-residues) of variant C, with 5P, 6P and 7P, respectively, described in *C*. *dromedarius*. While cryptic splice site isoforms (ΔQ83) of variant C, with different phosphorylation levels, were not found in the milk sample shown at Fig 4, they were successfully found in several milk samples. Whereas, α_s1_-CN short isoform was systematically present in all camel milk samples with 5, 6 and 7P (Table 3), by contrast, α_s1_-CN short isoforms of variant C occurred in some milk samples with 4P (24,611 Da) and up to 9P (24,929 Da). Herein, α_s1_-CN short isoforms of variants A and C carrying 6P groups are isoforms with the highest mass signal intensity values 50,634 *vs*. 47,392, respectively.

**Table 3:** Identification of camel milk protein (hybrid from Kyzylorda region) from observed molecular masses using LC-ESI-MS.

Peak	Ret.Time, min	Observed M_*r*_, Da	TheoreticalM_*r*_, Da	Protein description	UniProt/ NCBI GenBank Accession number	Intensity
I	4.50	21,157	21,158	κ-CN A, 1P, (GaN-Ga-SA2)x3*, pyro-E		1,361
		**21,184**	**21,182**	κ-CN B, 0P, (GaN-Ga)x3 + (GaN-Ga-SA2)x2**, pyro-E		**5,810**
II	18.61	18,210	18,210	κ-CN B, 0P ?	L0P304	161
		18,236	18,235	κ-CN A, 0P, pyro-E	P79139	72
III	24.32	12,564	12,564	WAP, 0P	P09837	1,756
		12,644	12,644	WAP, 1P		1,575
IV	24.97	23,878	23,878	α_s1_-CN A—short isoform (Δex 16 and Δex 13’), 4P		242
V	26.23	24,547	24,547	α_s1_-CN C -short isoform (Δex 16), 5P, splice variant (ΔQ83)		4,885
		**24,627**	**24,627**	**α**_**s1**_**-CN C—short isoform (**Δ**ex 16), 6P, splice variant (**Δ**Q83**		**21,606**
		24,707	24,707	α_s1_-CN C—short isoform (Δex 16), 7P, splice variant (ΔQ83)		6,990
		24,675	24,675	α_s1_-CN C—short isoform (Δex 16), 5P		9,441
		**24,755**	**24,755**	**α**_**s1**_**-CN C—short isoform** (Δex 16)**, 6P**	**K7DXB9**	**47,392**
		24,835	24,835	α_s1_-CN C—short isoform (Δex 16), 7P		7,046
		24,689	24,689	α_s1_-CN A—short isoform (Δex 16), 5P		9,748
		**24,768**	**24,769**	**α**_**s1**_**-CN A—short isoform** (Δex 16)**, 6P**	**O97943-2**	**50,634**
		24,849	24,849	α_s1_-CN A—short isoform (Δex 16), 7P		6,909
VI	28.53	**14,430**	**14,430**	**α-LAC**	**P00710**	**17,797**
		22,939	n/a	Uncharacterized protein 1 (UP1)	n/a***	2,701
		23,020	n/a	UP1+80Da	n/a	2,489
		23,099	n/a	UP1+160Da	n/a	1,079
		25,646	25,645	α_s1_-CN C, 6P, splice variant (ΔQ83)		3,501
		25,693	25,693	α_S1_-CN C, 5P		564
		**25,773**	**25,773**	**α**_**s1**_**-CN C, 6P**		**7,880**
		**25,787**	**25,787**	**α**_**s1**_**-CN A, 6P**	**O97943-1**	**3,472**
VII	30.05	21,825	21,826	α_s2_-CN, 7P		552
		**21,906**	**21,906**	**α**_**s2**_**-CN, 8P**	**O9794**	**5,242**
		21,984	21,986	α_s2_-CN, 9P		403
		23,178	n/a	UP1+240Da	n/a	1,256
VIII	31.11	21,986	21,986	α_s2_-CN, 9P	O97944	356
		**22,066**	**22,066**	**α**_**s2**_**-CN, 10P**		**4,790**
IX	33.18	22,066	22,066	α_s2_-CN, 10P		148
		**22,145**	**22,146**	**α**_**s2**_**-CN, 11P**		**1,964**
X	35.05	22,226	22,226	α_s2_-CN, 12P		894
		23,046	n/a	Uncharacterized protein 2 (UP2)	n/a	231
X	37.16	**19,143**	**19,143**	**PGRP**	**Q9GK12**	**7,207**
		23,206	n/a	UP2+160Da	n/a	1,592
		23,286	n/a	UP2+240Da	n/a	735
XII	38.09	66,481	66,477	CSA ?	XP_010981066.1	1,096
			66,491	LPO ?	Q9GJW6	
		66,512	n/a	CSA ? LPO?		2,663
		67,342	n/a	CSA ? LPO?		1,010
XIII	40.67	24,746	24,745	β-CN A, 3P, splice variant (ΔQ29)		2,073
		24,793	24,792	β-CN A, 2P		5,469
		24,825	24,825	β-CN A, 4P, splice variant (ΔQ29)		9,586
		24,873	24,872	β-CN A, 3P		10,177
		**24,953**	**24,953**	**β-CN A, 4P**	**A0A077SL35**	**84,494**
		24,842	24,842	β-CN B, 4P, splice variant (ΔQ29)		10,029
		24,891	24,890	β-CN B, 3P		10,365
		**24,970**	**24,971**	**β-CN B, 4P**	**Q9TVD0**	**87,973**
XIV	43.71	23,878	23,878	β-CN A-short isoform (Δ946 Da), 4P, splice variant (ΔQ29)		707
		23,963	23,958	β-CN A-short isoform (Δ946 Da), 5P, splice variant (ΔQ29)		244
		23,929	23,926	β-CN A-short isoform (Δ946 Da), 3P		438
		**24,006**	**24,006**	**β-CN A-short isoform (Δ946 Da), 4P**		**9,026**
		23,895	23,896	β-CN B-short isoform (Δ946 Da), 4P, splice variant (ΔQ29)		625
		**24,024**	**24,024**	**β-CN B-short isoform (Δ946 Da), 4P**		**5,545**
XV	47.02	12, 357	12,358	γ2-CN A, 0P		1,473
		12,376	12,376	γ2-CN B, 0P		1,065

Major proteins within each peak are in bold. Proteins and isoforms previously described are on grey background.

*(GaN-Ga-SA2) x 3, or (GaN-Ga) + (GaN-Ga-SA3) x 2, or (GaN-Ga-SA)+(GaN-Ga-SA2)+(GaN-Ga-SA3).

**(GaN-Ga) x 3 + (GaN-Ga-SA2) x 2, or (GaN-Ga) + (GaN-Ga-SA) x 4, or (GaN-Ga) x 2 + (GaN-Ga-SA) x 2 + (GaN-Ga-SA2), or (GaN-Ga) x 3 + (GaN-Ga-SA) + (GaN-Ga-SA3).

***n/a—not applicable.

Peak VI was more complex to interpret. Masses found in this peak belonged to four different molecular mass groups: 14,430 Da (ascribed to α-LAC), 22,939–23,099 Da (s.i. of 80 Da), 25,646 Da and 25,693–25,773 Da (s.i. of 80 Da), and 25,787 Da. Masses around 23 kDa (22,939–23,099 Da), with a mass increment of two P groups (160 Da), were not referenced to any protein in our database. These findings strongly suggest the existence of an additional uncharacterized phosphorylated protein, namely UP1, which remains to be identified. The third mass group, 25,646 Da and 25,693–25,773 Da, corresponds to a mixture of two long isoforms (214 and 215 aa residues, respectively) of α_s1_-CN variant C with 5P and 6P (25,693–25,773 Da) which differs from variant A by an aa substitution (E30D) in the mature protein [40]. The mass of 25,646 Da corresponds to a 214 aa residues isoform of α_s1_-CN variant C (ΔQ83), with 6P. The last molecular mass (25,787 Da) found in this peak was related to the mature variant A of α_s1_-CN bearing 6P groups, which is by far much less abundant than the short α_s1_-CN A-6P isoform (intensity of the mass signals: 3,472 *vs*. 50,634).

The four subsequent peaks (VII, VIII, IX, and X) all contained α_s2_-CN molecules, with phosphorylation levels ranging between 7P (21,825 Da, peak VII) and 12P (22,226 Da, peak X). Observed molecular masses of 21,825–21,984 Da were in perfect concordance with those predicted for α_s2_-CN displaying 7P and 9P, whereas α_s2_-CN with 8P (21,906 Da) was the most frequent isoform. In addition, the mass of 23,179 Da in peak VII probably corresponds to the UP1 found in fraction VI with one more P group. Masses ranging between 21,986 and 22,226 Da (s.i. of 80 Da) found in peaks VIII, IX, and X were related to α_s2_-CN variant A with 9P to 12P. These results suggest three more potential phosphorylation sites than reported by Kappeler *et al*. [38] who mentioned a maximum of 9 S residues phosphorylated in camel α_s2_-CN. More recently, Felfoul *et al*. [37] detected two α_s2_-CN isoforms with 10 and 11P groups in camel milk. Interestingly, peak X contains a second uncharacterized protein (UP2) with a molecular mass of 23,046 Da, not referring to any mass in our database for camel milk proteins. Such a mass was found in all camel milk samples analyzed so far (n = 30). This suggests the possible existence of a further phosphoprotein in camel milk, very likely a CN, since two putative related isoforms with two (23,206 Da) and three (23,286 Da) additional P groups were detected in peak XI, in which the most abundant mass found (19,143 Da) was attributed to PGRP.

In the hybrid from Kyzylorda region (Table 3), masses found in peak XII ranged between 66,481 and 67,342 Da. The most abundant masses 66,481 Da and 66,512 Da might be related to CSA of which the theoretical mass (peptide sequence predicted from the *C*. *dromedarius* genome, NCBI Accession number XP_010981066.1) is 66,477 Da. The mass differences of 4 Da and 35 Da could be attributed to putative genetic polymorphisms. The molecular weight reported by Felfoul *et al*. [37] from fresh camel milk was estimated as 66,600 Da. However, one cannot exclude that such masses could correspond to LPO depending on cleavage sites of the propeptide, when comparing with bovine LPO and human myeloperoxidase [41].

Molecular masses of 24,793–24,953 Da (s.i. of 80 Da) found in peak XIII, were ascribed to β-CN variant A with 2P, 3P and 4P, first described in the *C*. *bactrianus*. Molecular masses of 24,891–24,970 Da, which differ from β-CN A-3P and 4P by a 18 Da, correspond to β-CN variant B, first described in *C*. *dromedarius*. The mass difference of 18 Da between variants A and B is due to the M186I substitution. Isoforms of β-CN with 4P predominate whatever the milk sample and the genetic variant were, with equivalent intensity values of the mass signal for variants A and B, exemplified by a heterozygous hybrid camel: 84,494 *vs*. 87,973, respectively. In addition, the molecular mass of 24,842 Da, observed in peak XIII, corresponds to a splicing variant of β-CN B-4P. Such an isoform, which was so far considered as typical to the dromedary camel, was also found in hybrids and Bactrian camels. It is due to a cryptic splice site usage leading to the loss of the first codon (CAG) of exon 6, encoding residue Q29 in the protein.

Surprisingly, in the next peak (XIV), molecular masses around 24,000 Da (23,878 Da to 24,024 Da) were observed. Given the elution time and the mass range, these masses were very likely relative to the β-CN fraction, especially since a 18 Da mass differential existing between the pair of molecular masses (24,006 Da and 24,024 Da), is consistent with the occurrence of β-CN variants A and B, in both species. The important mass reduction, - 946 Da, relatively to the full-length β-CN, is hypothesized to be due to the cleavage by plasmin of the first seven N-terminal residues (1REKEEFK7) of the mature protein, given that this heptapeptide accounts for 947 Da. Furthermore, molecular masses equivalent to 23,878 Da and 23,895 Da are supposed to originate in the cryptic splice site usage (ΔQ29), previously mentioned.

Finally, in the last peak (XV) mass values 12,357 Da and 12,376 Da again with the mass difference in 18 Da were observed. These masses correspond very likely to camel γ_2_-CN A and B (12,357 Da *vs*. 12,376 Da, respectively), which are degradation products of β-CN [42].

This extensive analysis shows that mass accuracy provided by LC-ESI-MS was effective to allow protein identification of most of the protein isoforms by comparison of masses observed experimentally to theoretical molecular masses, and sufficiently powerful to recognize post-translational modifications (PTM) such as phosphorylation of CN, as well as genetic variants and long and short isoforms due to splicing inaccuracies.

### Multiple spliced variants of CSN1S1

To confirm the occurrence of *CSN1S1* multiple splice variants, we took advantage of the possibility to extract RNA from milk fat globules to sequence PCR fragments of cDNA encoding α_s1_-CN. Three different *CSN1S1* transcripts were found in each species and both genetic variants A and C. The nucleotide sequence of the most frequent variant transcript was shown to be deleted of exon 16, encoding the octapeptide EQAYFHLE. Besides, we also observed an isoform displaying the same sequence in which the first codon of exon 11 was lacking. Finally, a full-length transcript including exon 16 and the first codon of exon 11 was also detected, at a lower concentration.

## Discussion

Given the growing interest in camel milk, due to the health potential of its bioactive components [43] and frequently reported high anti-microbial activity [44], over the past 20 years and even more during the last decade, the milk protein fraction of Camelids, from all around the world has been extensively investigated [10], [11], [36], [37], [39], [40], [45–53]. All these studies have explored, with more or less efficient approaches, the composition of the major milk proteins. However, the molecular diversity of these major proteins had not yet been studied. Then, our main objective was i) to provide, if not a comprehensive, at least an in-depth description of the protein fraction of camel milk; ii) to go further into an extensive analysis of the molecular diversity of major milk proteins from Camelids (*C*. *dromedarius*, *C*. *bactrianus*, and hybrids) sampled from different regions in Kazakhstan. For these purposes, different proteomic tools and methodological approaches were applied. For short, up to 391 protein species were identified in cumulating LC-MS/MS analyses of 8 individual *Camelus* milk, and the extensive characterization of CN and whey protein polymorphisms, using LC-ESI-MS, revealed a minimum of 50 molecular species.

### Interspecies in-depth proteomic analysis of camel milk proteins

To our knowledge, the number of proteins identified in this study was relatively higher compared to the numbers reported in previous studies on the camel proteome [10], [11]. The largest camel milk proteome determined so far comprised about 238 proteins including some known camel proteins and heavy-chain immunoglobulins [10]. In this study carried out on *C*. *dromedarius*, proteins were identified from 2D SDS-PAGE with subsequent matrix-assisted laser desorption/ionization (MALDI) time-of-flight mass spectrometry analysis. However, it should be mentioned that several of the 238 proteins identified matched with the same protein in different species. Hence, at most *ca*. 140 proteins may be considered as unique. By comparison, in the present study a total of 391 unique protein species were determined from LC-MS/MS analyses of *C*. *bactrianus* (n = 3), *C*. *dromedarius* (n = 3), and hybrids (n = 2), sampled from three different regions (Atyrau, Shymkent and Kyzylorda). Proteins such as flavin monoamine oxidase, perilipin 2, neutrophil gelatinase-associated lipocalin-like protein, brain-specific serine protease 4-like protein and others, which were not determined previously, were successfully detected. Conversely, about 30 proteins identified by Alhaider and co-workers [10] were not found in our study.

However, as for other mammals, CN represent the major protein fraction of camel milk (80%), among which β-CN is the most abundant [54]. Quantitative analyses performed by Kappeler *et al*. [35] on camel milk CN have demonstrated significant higher amounts of β-CN (15 g/L *vs*. 10 g/L) compared to the homologous bovine β-CN and significant lower amounts of κ-CN (0.8 g/L *vs*. 3.5 g/L). Regarding relative proportions, as previously reported [38], α_s1_-, α_s2_-, β- and κ-CN contribute to about 22%, 9.5%, 65%, and 3.5% of total CN, respectively. Taking into account the 30 milk samples analyzed in LC-ESI-MS, relative proportions of individual CN, estimated from the mass signal intensity of each CN family (summing the mass signal of its phosphorylation and splicing isoforms) relatively to the sum of mass signal intensities of all CN families (considering that ionizing properties of caseins and their isoforms are comparable), were 37% α_s1_-CN, 6.1% α_s2_-CN, 53.1% β-CN, and 3.8% κ-CN. These values varied considerably compared to those reported previously by Kappeler *et al*. [38] essentially as far as α_s1_-CN and β-CN are concerned. Whereas α_s1_-CN accounts for 36.1% for *C*. *bactrianus*, it reaches 37.4% and 37.6% in *C*. *dromedarius* and hybrids, respectively (Table 4). Percentage of α_s1_-CN calculated in our study was 15% higher than the value reported by Kappeler *et al*. Such an increase is compensated in part by a decrease of 12% of β-CN. The small amount of κ-CN observed is probably underestimated, since most of the highly glycosylated isoforms were not detected. However, this is in agreement with the fact that the size distribution of CN micelles is inversely related to κ-CN content [55], [56], since camel CN micelles are the largest, ranging in size between 280–550 nm [57].

**Table 4:** Relative proportion of each CN expressed in %, estimated from the mass signal intensity of each CN family relatively to the sum of mass signal intensities of all CN families in the three camel species.

	κ-CN		α_s1_-CN		α_s2_-CN		β-CN	
	m	σ	m	σ	m	σ	m	σ
**Bactrian**	3,09	1,89	36,09	2,33	7,13	1,49	53,68	2,08
**Dromedary**	3,63	2,13	37,39	3,89	5,79	0,98	53,19	3,46
**Hybrid**	4,77	3,01	37,57	3,03	5,25	1,56	52,41	4,18

m = mean.

σ = standard deviation.

Even though, there are 2 potential phosphorylation sites in κ-CN (S141 and S159) conserved and phosphorylated in sheep and goats [27] only isoforms with a single or no P group in the first chromatographic peak comprising glycosylated isoforms with 3 or 5 carbohydrate motifs were detected. Five glycosylated isoforms of camel κ-CN ranging in size between *ca*. 24 and 25.9 kDa were found in camel milk using 2D SDS-PAGE [50].

In addition, γ_2_-CN, a C-terminal product resulting from a highly specific proteolysis of β-CN by the natural milk protease (plasmin) was successfully found in the milk samples analyzed. Previously published data suggested that the proportion of γ-CN in total CN fraction is highest at the beginning and the end of lactation, and in very low yielding animals [56]. The molecular masses observed in this study (12,357 Da and 12,376 Da) were lower from those previously observed by Kappeler [38]: 13.9 kDa, 15.7 kDa and 15.75 kDa.

Immune-related proteins such as GlyCAM1, MFGE8 and LTF were detected in camel milk. GlyCAM1, also named lactophorin or PP3 is a cysteine free protein, which belongs to the family of GlyCAM-type molecules [58]. Two splicing variants A and B were distinguished in camel milk [47]. Variant A encoding 137 aa residues has a M_*r*_ of 15.7 kDa, while variant B encoding 122 aa residues has a M_*r*_ of 13.8 kDa. The primary structure of Variant A reveals 54% identity with a protein isolated from bovine milk [59]. Until late, it has been claimed that camel GlyCAM1 is neither glycosylated nor phosphorylated as bovine GlyCAM1. However, Girardet *et al*. [22] suggested the probable existence of one O-glycosylation site (16TDT18) in variant A of which the apparent M_*r*_ was estimated as 22.5 kDa from SDS-PAGE. Using the same approach, two bands were found, in which we identified GlyCAM1 from LC-MS/MS analysis 22 kDa and 10 kDa, corresponding probably to the glycosylated and putatively phosphorylated isoform of GlyCAM1 observed by Girardet *et al*. [22], and to a product of proteolysis, respectively. Surprisingly, no molecular masses corresponding to camel GlyCAM1 A and B were identified by LC-ESI-MS analysis. Likewise, LC-ESI-MS did not permit to detect LTF, even though, SDS-PAGE and LC-MS/MS data confirm its presence in analyzed camel milk samples. On the other hand, molecular masses ranging between 74,338 Da-79,621 Da could be attributed to camel LTF of which the theoretical mass reported by Kappeler *et al*. [60] for the mature protein (689 aa residues long) without PTM is 75,250 Da. Therefore, the mass difference observed is very likely attributable to PTM. In addition, Konuspayeva *et al*. [61] reported that the level of LTF is affected by seasonal variations.

Elsewhere, MFGM-enriched proteins such as XO, BTN, fatty acid synthase, actin, ras-related protein Rab-18, ADP-ribosylation factor 1, tyrosine-protein kinase, GTP-binding protein SAR1b were identified in Kazakh camel milk samples in accordance with previous results obtained with *C*. *dromedarius* [62] and *C*. *bactrianus* [11] milk samples. Surprisingly, whereas BTN was present in all milk samples, it seems to be absent in *C*. *bactrianus* from Atyrau region. This could be due to the way the band in the electrophoresis gel was cut, since BTN was found in the other seven samples analyzed. Regarding proteins originating from blood, such as serpin A3-1, apolipoprotein A-1, α-1-antitrypsin like protein, α-1-acid glycoprotein, β-2-microglobulin, complement C3-like protein, they were also found in Kazakh camel milks, in agreement with findings of Yang *et al*. [11] reported for Bactrian camels from China. By contrast, as mentioned in the Results section, no trace of CSA was found in Kazakh milk samples from LC-MS/MS analyses, whereas its presence is suspected from LC-ESI-MS.

A heat shock protein (HSPA6 also called HSP70B’) occurred at rank 23 amongst the first third of the most represented proteins in Kazakh camel milks (Table 2). Expression of heat shock proteins, including HSP70 is increased during heat stress and involved in defense against dehydration or thermal stress in arid environments [63], [64]. The entire sequence of this protein has been deduced from the nucleotide sequence of a full-length cDNA in *C*. *dromedarius* [65]. Comprising 643 aa residues, the camel protein, of which the M_*r*_ is 70,543 Da in agreement with the molecular mass estimated from SDS-PAGE, shares a high similarity (94% identity) with cow and pig HSP70.

Against all expectations, peptides with sequence similarity with bovine *β*-lactoglobulin, the major allergen in bovine milk, were identified in the 8 camel milk samples (Bactrian, dromedary and hybrids) from Kazakhstan, analyzed by LC-MS/MS. The coverage percentage ranged between 30 and 60% in individual milk samples, and reached 71% cumulating all the peptides found. Five peptides related to bovine *β*-lactoglobulin were also detected by Alhaider *et al*. [10] in camel milk from Saudi Arabia and the United States. Youcef *et al*. [48] revealed a weak cross reaction between dromedary whey proteins and IgG anti bovine *β*-lactoglobulin. Such findings disagree with the usually admitted notion that *β*-lactoglobulin is absent in camel milk [50], [66]. Even though we cannot exclude a possible contamination by bovine milk (unlikely with the 8 camel milk samples analyzed by LC-MS/MS) or the presence in camel milk of a Progesterone Associated Endometrial Protein (PAEP) displaying strong similarities with *β*-lactoglobulin. However, significant similarities between human PAEP and the peptides having allowed the identification of *β*-lactoglobulin in *C*. *bactrianus* milk, were not found.

### Molecular diversity of camel caseins: Genetic polymorphism and alternative splicing

Regarding camel α_s1_-CN, the situation is particularly confusing. Kappeler *et al*. [38] first described two cDNA (short and long), encoding two protein isoforms of 207 and 215 aa, named A and B variants. The A variant corresponds to the short isoform (207 aa), in which the octapeptide 155EQAYFHLE162 encoded by exon 16 was missing, whereas this octapeptide is present in the 215 aa-long isoforms. In our study, two isoforms long and short showing a 1,018 Da mass difference were found, in which the short isoform was the major component (*ca*. 90%) of total camel α_s1_-CN. Such an alternative splicing event has been first reported in goats [67], sheep [68], [69] and later in lama [70]. In addition, we observed the existence of two distinct genetic variants called A and C, arising from the E30D aa substitution, as previously reported by Shuiep *et al*. [39]. Since, variants A and B described by Kappeler *et al*. [38] displayed a E aa residue in position 30 of the mature peptide chain, it becomes obvious that Kappeler’s A and B variants derived in fact from a single allele, of which the primary transcript is subject to exon 16 skipping during the splicing process. In other words, the B variant is nothing other than a splicing variant of a single allele that we propose to call CSN1S1*A.

Recently, Erhardt *et al*. [40] reported in *C*. *dromedarius* from different regions of Sudan, the existence of a further variant, called D, clearly displaying a different IEF behavior. Excluding this D variant, which was not precisely characterized, there are α_s1_-CN long and short non-allelic isoforms arising from alternative splicing of a single primary transcript and only two perfectly characterized genetic variants A and C resulting from a single G>T nucleotide substitution in exon 4 and leading to E30D aa substitution. This molecular diversity is becoming more complex due to different phosphorylation levels ranging between 5-8P groups (see thereafter) and due to isoforms arising from cryptic splice site usage [67], [71], [72], leading to the loss of a Q residue corresponding to the first codon of exon 11. Results from cDNA sequencing substantiate this.

Electrophoretic and LC-MS analyses as well as cDNA sequencing confirmed that β-CN occurs as two genetic variants A and B, with the aa substitution M186I (yielding a -18 Da mass difference). The most frequent form of β-CN had 4P groups, one P group more than reported for Somali, Turkana and Pakistani camels by Kappeler *et al*. [38]. Surprisingly, in Kazakh populations, a second series of β-CN components with lower molecular masses (mass difference: -946 Da), relatively to the full-length β-CN were found. This phenomenon, observed with both genetic variants, might be due to the cleavage by plasmin of the first seven N-terminal residues (REKEEFK) of the mature protein. A mass difference of 947 Da was observed between the native full-length protein with 4P (24,953 Da and 24,971 Da for A and B variants, respectively) and the plasmin cleavage product at the same phosphorylation level (24,006 Da and 24,024 Da for A and B variants, respectively). The occurrence of a K residue in position 7 of the mature β-CN does not occur in any other species, of which the N-terminal sequence is known [27]. However, our results strongly suggest that the peptide bond 7K-T8 is sensible to plasmin that is, like trypsin, a serine protease. Indeed, REKEEFK was present amongst tryptic peptides identified in LC-MS/MS analysis.

There is another even less probable possibility, involving the deletion of exon 5 that encodes 8 aa residues (ESITHINK for a mass of 923 Da), since a similar event was previously characterized from mare [73] and donkey [74] milks. However, sequencing of camel β-CN cDNA has not revealed any deletion in the mRNA encoding this protein (results not shown), consistently with Kappeler *et al*. [38] who only reported a full-length sequence for β-CN, conversely to α_s1_-CN. Since in our study we were not able to provide any further confirmation of the presence of shorter mRNA of camel β-CN in which exon 5 is spliced out, we give preference to the cleavage by plasmin of the first seven N-terminal residues of β-CN rather than an alternative splicing process.

Surprisingly, two so far uncharacterized proteins (UP1 and UP2) with molecular masses around 23,000 Da and different phosphorylation levels were observed, suggesting they are possibly proteins related to CN. However, to prove this hypothesis further research for in depth characterization of these proteins is necessary.

### Post-translational modifications of milk proteins: Phosphorylation of caseins

Among the various approaches developed in proteomics, electrospray ionization (ESI) mass spectrometry (MS) is eminently suitable for studying PTM, including phosphorylation and glycosylation, since the technique provides molecular mass determination of native proteins. Phosphorylation of proteins is one of the most frequent PTM in eukaryotic cells. It has become a common knowledge that phosphorylation of CN occurs at S or T aa residues in tripeptide sequences S/T-X-A where X represents any aa residue and A is an acidic residue [75]. This consensus sequence is recognized by FAM20C, a Golgi CN-kinase, which phosphorylates secreted phosphoproteins, including both CN and members of the small integrin-binding ligand N-linked glycoproteins (SIBLING) protein family, which modulate biomineralization [76]. Each phosphorylation event adds 79.98 Da to the molecular mass of the peptide chain [77]. It was predicted with high confidence 8 probably phosphorylated S residues in α_s1_-CN (S18, S68, S70, S71, S72, S73, S193, and S202), 9 potential phosphorylated S residues in α_s2_-CN (S8, S9, S10, S32, S53, S108, S110, S113, and S121), 4 S residues in β-CN (S15, S17, S18, and S19), and 2 S residues in κ-CN (S141 and S159). However, up to 9P residues per α_s1_-CN molecule were observed whatever the genetic variant is. Theoretically, given the S/T-X-A consensus rule, there are 4 T residues that could be phosphorylated (T55, T80, T153, and T196), leading to a maximum of 12 P groups per molecule. Therefore, we can put forward that at least one of the four T residues is phosphorylated in the α_s1_-CN-9P.

**Fig 5:**
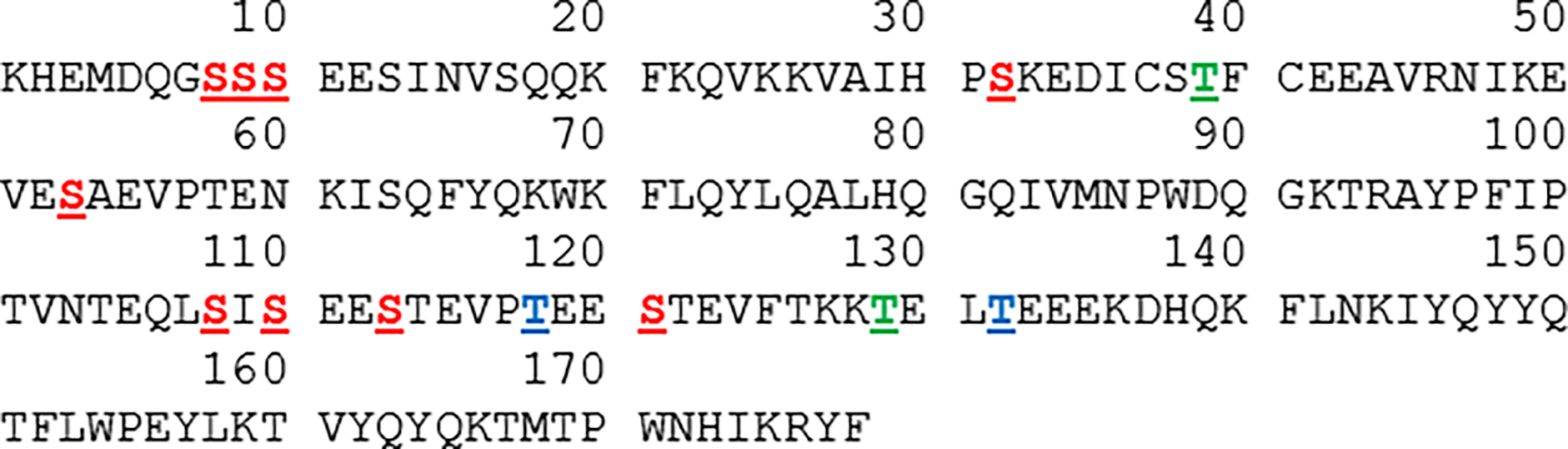
Amino acid sequence of mature camel α_s2_-CN with potential phosphorylation sites. Seryl and Threonyl residues matching the S/T-X-A motif are in red and blue, respectively, and underlined. Threonyl residues matching the S/T-X-X-A motif are in green and underlined.

With 11 potentially phosphorylated aa residues matching the S/T-X-A motif (Fig 5), camel α_s2_-CN displays the highest phosphorylation level, in agreement with Felfoul *et al*. who reported recently 11P groups [37]. To reach such a phosphorylation level, besides the nine SerP, two putative ThrP (T118 and T132) have to be phosphorylated. In all the Kazakh milk samples analyzed in LC-ESI-MS we found α_s2_-CN with 12 P groups, as the molecular mass of 22,226 Da observed corresponds to the mass of the peptide backbone (21,266 Da) increased by 960 Da, a mass increment which coincides with 12 P groups. That means that at least another S/T residue that does not match with the canonic sequence recognized by the mammary kinase(s), is potentially phosphorylated. According to Allende *et al*. [78] the sequence S/T-X-X-A follow-through with the minimum requirements for phosphorylation by the CN-kinase II (CK2). It is critical to highlight in this regard that E or D in this site can be replaced by SerP or ThrP. Two T residues, namely T39 and T129 in the camel α_s2_-CN fully meet the requirements of the above-mentioned motif (Fig 5) and could be phosphorylated. Such an event is the only hypothesis to reach 12P for camel α_s2_-CN. Since these two kinases are very likely secreted, the idea that phosphorylation at T39/T129 may occur in the extracellular environment cannot be excluded. This warrants further investigation. Fam20C, which is very likely the major secretory pathway protein kinase [79], might be responsible for the phosphorylation of S and T residues within S/T-X-A motif, whereas a CK2-type kinase might be responsible for phosphorylation of T residue within an S/T-X-X-A motif. This is in agreement with the hypothesis put forward by Bijl *et al*. [80] and Fang *et al*. [81], who suggest from phenotypic correlations and hierarchical clustering the existence of at least 2 regulatory systems for phosphorylation of α_s_-CN. Elsewhere, bovine milk osteopontin which is a multiphosphorylated glycoprotein also found in bone, was shown to contain 27 SerP and one ThrP [82]. Twenty five SerP and one ThrP were located in S/T-X-E/S(P)/D motifs, whereas two SerP were found in the sequence S-X-X-E/S(P).

## Conclusions

In this study, six main findings combining proven proteomic and molecular biology approaches are provided. The first one is an enhancing of our knowledge of camel milk protein composition. The second one is deciphering the extreme complexity of camel CN fraction due to PTM (phosphorylation) and splicing events (exon skipping and cryptic splice site usage). The third finding is the detection of two unknown proteins, UP1 and UP2 that remain to be characterized. In addition, we provide results substantiating the possible existence of a camel β-lactoglobulin. However, this result requires further investigation, currently in progress in the laboratory. Afterwards, we report for the first time the presence of α_s2_-CN-12P, and short isoforms of β-CN probably arising from proteolysis by plasmin, the natural protease of milk. The ultimate finding is the demonstration that genetic variants, which hitherto seemed specific to a species (β-CN A for Bactrian and β-CN B for dromedary), are in fact present in both *dromedarius* and *bactrianus*.

## Supporting information

S1 Table Functional groups of proteins identified in camel milk by LC-MS/MS. (XLSX)

S1 Text Permission to publish content under CC-BY license. (PDF)
