# Supplementary material for: Combining different proteomic approaches to resolve complexity of the milk protein fraction of dromedary, Bactrian camels and hybrids, from different regions of Kazakhstan
Source: PLoS One. 2018 May 10;13(5):e0197026. doi: 10.1371/journal.pone.0197026 (PMC5944991; doi:10.1371/journal.pone.0197026)
Supplement: S1 Text — (PDF) [file pone.0197026.s002.pdf]

Request for Permission to Publish Content under CC-BY License

Dear Rights Holder or Representative,

I have submitted a paper for publication in a PLOS journal, and wish to include the content listed below in the paper. I'm hereby requesting your (or your company's or institution's) permission to include the content in my paper. Please note that all PLOS journals are published under a Creative Commons Attribution License (CC BY), which allows for unrestricted use and distribution, even commercial, as long as attribution is given to the creator or rights holder of the content. See https://creativecommons.org/licenses/by/4.0/.

To grant me permission to use the content in my PLOS paper, please fill in the information below and then scan the completed form and send it to me at my email address.

Thank you.

My name:

Alma Ryskaliyeva

My email address:

alma.ryskaliyeva@inra.fr

Description of the content which I'm seeking permission to use (citation and/or title, and pasted screen shot, if applicable):

I request permission for the open-access journal PLOS ONE to publish photos taken in Kazakhstan under the Creative Commons Attribution License (CCAL) CC BY 4.0 (http://creativecommons.org/licenses/by/4.0/). Please be aware that this license allows unrestricted use and distribution, even commercially, by third parties. Please reply and provide explicit written permission to publish photos under a CC BY license.

Link to the Content:

http://camelides.cirad.fr/fr/science/pdf/presentation_these_konuspayeva.pdf

* * *

On behalf of myself or the rights holder, I hereby grant the permission sought herein.

Signature of Party Granting Permission:

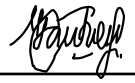

Date:

06/03/2018

Printed Name and Title:

Dr Gaukhar Konuspayeva
